# Quantitative Comparison of Yield, Quality, and Metabolic Products of Different Medicinal Parts of Two Types of *Perilla frutescens* Cultivated in a New Location from Different Regions

**DOI:** 10.3390/plants14101486

**Published:** 2025-05-15

**Authors:** Zhenbin Huang, Xiang Zhang, Liangshuai Fan, Xiaojun Jin, Hongyan Wang, Jiali Cheng, Chenyue Wang, Qing Fang

**Affiliations:** 1 College of Agronomy, Gansu Agricultural University, Lanzhou 730070, China; huangzhenbin1111@163.com (Z.H.); 1073323120618@st.gsau.edu.cn (X.Z.); 1073323120614@st.gsau.edu.cn (L.F.); wanghy@st.gsau.edu.cn (H.W.); chengjl@st.gsau.edu.cn (J.C.); wcy04042023@163.com (C.W.); 107332202243@st.gsau.edu.cn (Q.F.); 2 State Key Laboratory of Arid Habitat Crops, Gansu Agricultural University, Lanzhou 730070, China

**Keywords:** *Perilla frutescens*, leaf color, yield quality, metabolic products

## Abstract

This study focuses on multiple origins of green-back purple and dual-faced purple *Perilla frutescens*, employing field cultivation experiments combined with detection methods, such as HPLC, LC-MS, and GC-MS, to compare the differences in yield, quality, and metabolic products of the different colored *P. frutescens*. The results indicate that green-back purple *P. frutescens* significantly outperformed dual-faced purple *P. frutescens* in terms of leaf, stem, and seed yields, while the effective component contents in the leaves and seeds of dual-faced purple *P. frutescens* are higher than those of dual-faced green *P. frutescens*. An analysis of the anthocyanin components in *P. frutescens* leaves and the volatile components in *P. frutescens* seeds shows that the total anthocyanin content in dual-faced purple *P. frutescens* leaves is 34.63% higher than that in green-back purple *P. frutescens*, whereas the total volatile components in the seeds of green-back purple *P. frutescens* exceeds those in dual-faced *P. frutescens* by 12.99%. The Mantel test indicates a potential correlation mechanism between the anthocyanin components in *P. frutescens* leaves and the volatile components in *P. frutescens* seeds, which are significantly associated with the yield quality of both *P. frutescens* leaves and seeds. This study found that *P. frutescens* with blue–green leaves yields more than double-sided purple *P. frutescens*, although the quality of its leaves and seeds is inferior to that of double-sided purple *P. frutescens*. Furthermore, the anthocyanin components in *P. frutescens* leaves and the volatile components in *P. frutescens* seeds exhibit significant correlations with the yield and quality of both leaves and seeds, offering important insights for the production and application of *P. frutescens*.

## 1. Introduction

*Perilla frutescens* (L.) Britt. is an annual, short-day herb belonging to the Lamiaceae family. It is widely distributed and extensively cultivated, especially in Asian countries, such as China, Japan, Korea, and Vietnam [1,2]. *P. frutescens* is consumed as both a vegetable and a spice, and it is commonly employed in traditional herbal medicine to treat various ailments. The leaves are used in salads, seasonings, and food colorants, and they possess pharmacological effects, including antioxidant, anti-tumor, and lipid-lowering properties. [3,4,5]. *P. frutescens* stems are utilized to treat vomiting and abdominal pain, as well as to prevent miscarriage, while *P. frutescens* seeds exhibit antitussive, expectorant, and antipyretic effects. Additionally, *P. frutescens* seeds are recognized as an important traditional oilseed crop [6,7,8]. Because of its substantial nutritional and medicinal value, *P. frutescens* has become an integral component of daily life and has garnered increasing attention [6,9].

**Figure A1:**
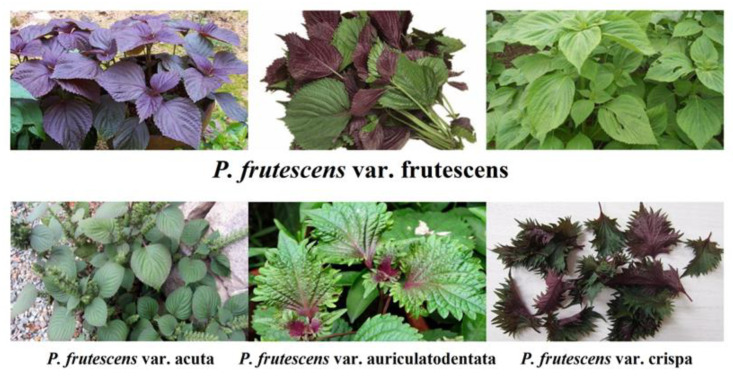
Morphological appearance of plants in the *P. frutescens* genus.

According to the “Flora of China”, Perilla is classified into three varieties of the species *P. frutescens,* differing significantly in leaf color. P. frutescens can be classified into three types based on leaf color: green on both sides, green on the upper side with a purple underside, and purple on both sides (Figure A1) [6]. Significant differences in chemical compositions exist among the varieties of *P. frutescens*. For instance, the concentrations of four flavonoids, two organic acids, and one coumarin are substantially higher in red-leaved *P. frutescens* compared with green-leaved *P. frutescens* [10]. Dossou et al. found that both leaf color and seed coat color influence the phytochemical characteristics and antioxidant activity of *P. frutescens* seeds [11]. This finding suggests significant differences among *P. frutescens* plants with varying leaf colors. Although all aerial parts of *P. frutescens* are utilized, research predominantly focuses on its leaves [12,13]. Research on *P. frutescens* seeds primarily concentrates on oil yield, physicochemical properties, and composition. Lee et al. found that superheated steam (SHS) treatment significantly increases the oil yield of *P. frutescens* seeds without generating characteristic odor compounds [14]. Moreover, Guan et al. proposed that rosmarinic acid, luteolin, apigenin, and rosmarinic acid-3-O-glucoside serve as chemical markers for assessing *P. frutescens* seed quality [15].

While previous studies have explored the nutritional components and medicinal value of *P. frutescens*, comprehensive research examining the relationship between leaf color variations, plant growth, yield quality, and their metabolic products remains limited. This study systematically compares the growth conditions and metabolic products of green-backed purple (L1) and double-sided purple (L2) *P. frutescens* from five distinct production areas (P1-P5) for the first time. By integrating field cultivation techniques with analytical methods, such as HPLC, LC-MS, and GC-MS, we thoroughly investigate the effects of different leaf colors on *P. frutescens*, thus providing new theoretical foundations and practical insights for industry development. Additionally, this research employs Mantel tests and Pearson correlation analyses to examine the relationship between metabolic products and yield quality—an area that has not received adequate attention in the existing literature. Consequently, the novelty of this study lies in its ability to reveal the unique characteristics of *P. frutescens* with varying leaf colors and their potential impact on economic value.

## 2. Results

### 2.1. Yield and Quality of P. frutescens

#### 2.1.1. Yield and Quality of *P. frutescens* Leaves

**Figure 1:**
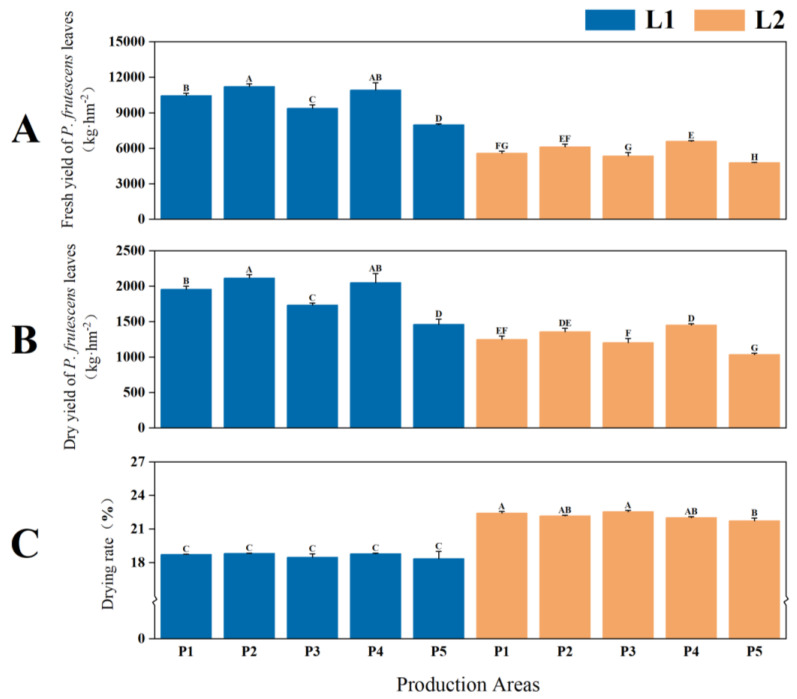
Differences in the fresh yields (**A**), dry yields (**B**), and dry weight ratios (**C**) of the leaves of two *P. frutescens* types from different origins. L and P represent the leaf color and origin of the *P. frutescens*, respectively. Different uppercase letters indicate highly significant differences among various treatments (*p* < 0.01), and this convention applies throughout the text.

Highly significant differences in leaf yield and dry weight ratio were observed between the two types of *P. frutescens* with different leaf colors (*p* < 0.01), with the L1 leaf color *P. frutescens* exhibiting a higher yield than L2. The fresh yield of the L1 leaf color *P. frutescens* was 9985.30 kg·hm^−2^ (Figure 1A), while the dry yield was 1861.69 kg·hm^−2^ (Figure 1B), which were 4304.16 kg·hm^−2^ and 602.47 kg·hm^−2^ higher than those of L2, respectively. The dry weight ratio of the L1 leaf color *P. frutescens* was 18.62% (Figure 1C), which is 3.54% lower than that of L2. Overall, the highest yield of *P. frutescens* leaves was recorded for the L1 leaf color *P. frutescens* from the P2 location, with a fresh yield of 11,217.34 kg·hm^−2^ and a dry yield of 2111.11 kg·hm^−2^, while the highest dry weight ratio, at 22.52%, was found in the L2 *P. frutescens* from the P3 location.

**Figure 2:**
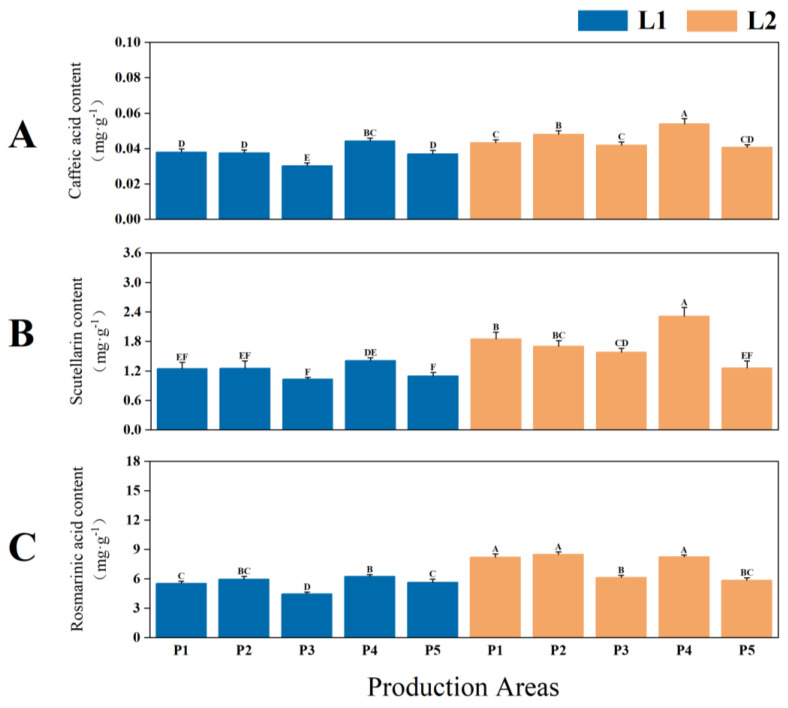
Differences in the contents of caffeic acid (**A**), scutellarin (**B**), and rosmarinic acid (**C**) in the leaves of two types of *P. frutescens* from different origins. L and P represent the leaf color and origin of the *P. frutescens*, respectively. Different uppercase letters indicate highly significant differences among the various treatments (*p* < 0.01).

Significant differences in the contents of effective components were observed in the leaves of two types of *P. frutescens* with different leaf colors (*p* < 0.01), with the quality of the L2 leaf color *P. frutescens* being superior to that of L1. The content of caffeic acid in the L1 leaf color *P. frutescens* was 0.0375 mg·g^−1^ (Figure 2A), while the contents of scutellarin and rosmarinic acid were 1.2080 mg·g^−1^ (Figure 2B) and 5.5677 mg·g^−1^ (Figure 2C), respectively—lower than those of L2 by 0.0081 mg·g^−1^, 0.5346 mg·g^−1^, and 1.8263 mg·g^−1^. Overall, the highest content of effective components in *P. frutescens* leaves was found in the L2 *P. frutescens* from the P4 location, with the caffeic acid content measuring 0.0540 mg·g^−1^, scutellarin content at 2.3170 mg·g^−1^, and rosmarinic acid content at 8.2417 mg·g^−1^.

#### 2.1.2. Yield and Quality of *P. frutescens* Stems

**Figure 3:**
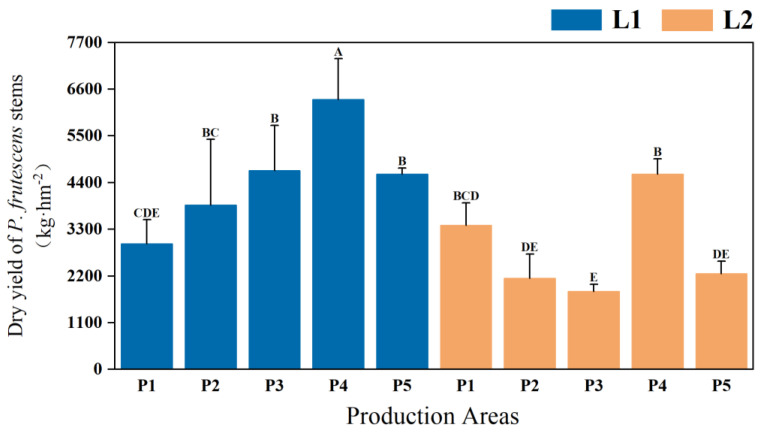
Differences in stem yield of two types of *P. frutescens* from different origins. Different uppercase letters indicate highly significant differences among the various treatments (*p* < 0.01).

Significant differences in stem yield were observed between the two types of *P. frutescens* with different leaf colors (*p* < 0.01), with the L1 leaf color *P. frutescens* demonstrating a higher yield than L2. The stem yield of the L1 leaf color *P. frutescens* was 4492.80 kg·hm^−2^ (Figure 3), which is 1646.22 kg·hm^−2^ greater than that of the L2 leaf color. Overall, the highest yield of *P. frutescens* stems was recorded for the L1 leaf color *P. frutescens* from the P4 location, measuring 6355.56 kg·hm^−2^.

**Figure 4:**
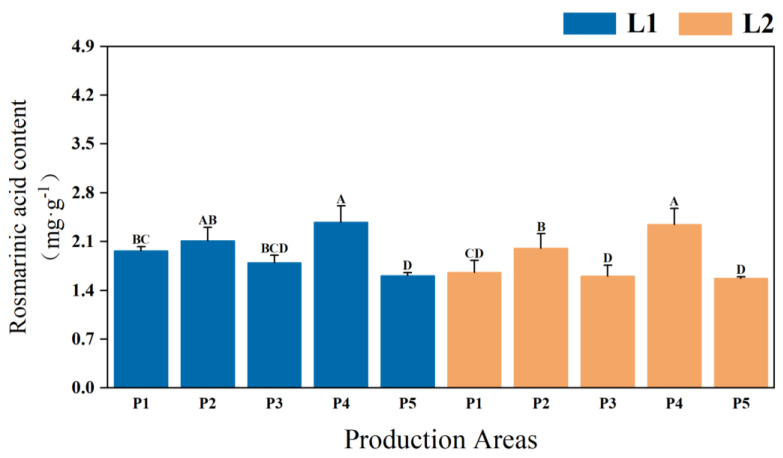
Differences in rosmarinic acid content were observed in the stems of two types of *P. frutescens* from different origins. Different uppercase letters indicate highly significant differences among the various treatments (*p* < 0.01).

Significant differences in the content of effective components were observed in the stems of two types of *P. frutescens* with different leaf colors (*p* < 0.01), with L1 leaf color *P. frutescens* stems exhibiting a higher quality than those of L2. The content of rosmarinic acid in the L1 leaf color *P. frutescens* stems was 1.9729 mg·g^−1^ (Figure 4), which is 0.1355 mg·g^−1^ greater than that of the L2 *P. frutescens* stems. Overall, the highest content of effective components in *P. frutescens* stems was recorded for the L1 *P. frutescens* from the P4 location, with rosmarinic acid content measuring 2.3757 mg·g^−1^.

#### 2.1.3. Yield and Quality of *P. frutescens* Seeds

**Figure 5:**
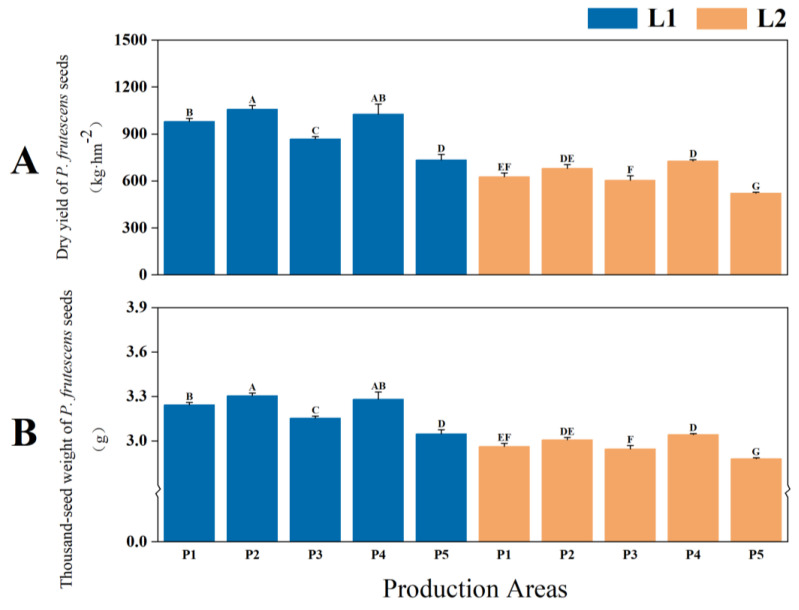
Differences in the seed yields (**A**) and thousand-seed weights (**B**) of two types of *P. frutescens* from different origins. Different uppercase letters indicate highly significant differences among the various treatments (*p* < 0.01).

Significant differences in seed yield and thousand-seed weight were observed between two types of *P. frutescens* with different leaf colors (*p* < 0.01), with L1 leaf color *P. frutescens* demonstrating higher yield and thousand-seed weight compared with L2. The seed yield of the L1 leaf color *P. frutescens* was 932.97 kg·hm^−2^ (Figure 5A), while the thousand-seed weight was measured at 3.2052 g (Figure 5B), which were 301.22 kg·hm^−2^ and 0.2389 g higher than those of L2, respectively. Overall, the highest yield and thousand-seed weight were recorded for the L1 leaf color *P. frutescens* from the P2 location, with a yield of 1057.68 kg·hm^−2^ and a thousand-seed weight of 3.3041 g.

**Figure 6:**
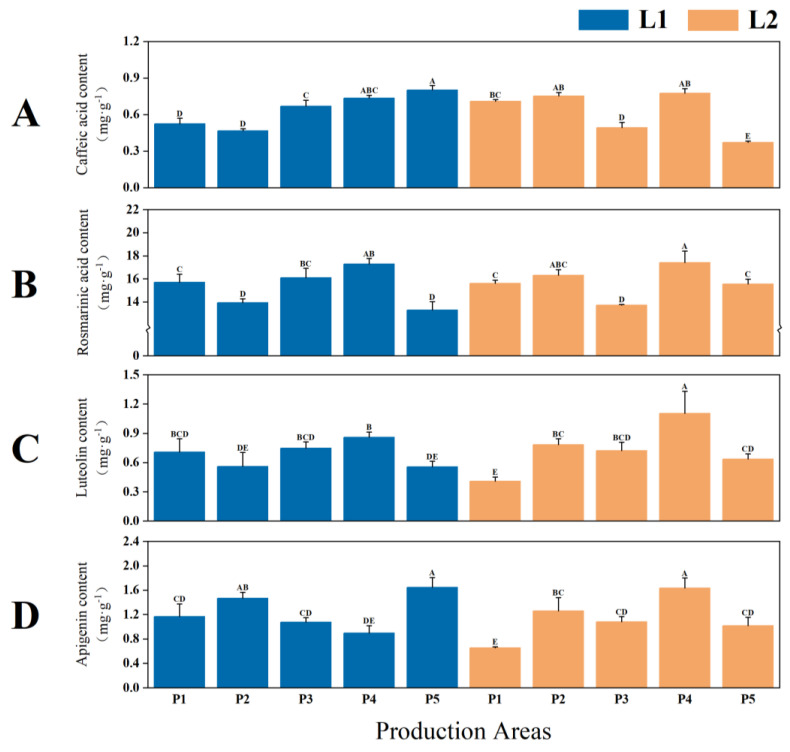
Differences in the contents of caffeic acid (**A**), rosmarinic acid (**B**), luteolin (**C**), and apigenin (**D**) in the seeds of two types of *P. frutescens* from different origins. Different uppercase letters indicate highly significant differences among the various treatments (*p* < 0.01).

There are highly significant differences in the contents of active ingredients in the seeds of two types of *P. frutescens* with distinct leaf colors (*p* < 0.01), with the L2 leaf-colored *P. frutescens* seeds exhibiting superior quality compared with the L1 variety. The caffeic acid content in the L1 leaf-colored *P. frutescens* seeds was 0.6395 mg·g^−1^ (Figure 6A), and the apigenin content was 1.2495 mg·g^−1^ (Figure 6D), which surpass those found in the L2 *P. frutescens* seeds by 0.0189 mg·g^−1^ and 0.1207 mg·g^−1^, respectively. In contrast, the rosmarinic acid content in the L1 leaf-colored *P. frutescens* seeds was 15.2597 mg·g^−1^ (Figure 6B), while the luteolin content was 0.6855 mg·g^−1^ (Figure 6C), which are lower than those of the L2 *P. frutescens* seeds by 0.4570 mg·g^−1^ and 0.0450 mg·g^−1^, respectively. Overall, the highest contents of active ingredients in the *P. frutescens* leaves was observed in the L2 variety from the P4 origin, with caffeic acid at 0.7763 mg·g^−1^, rosmarinic acid at 17.4113 mg·g^−1^, luteolin at 1.1030 mg·g^−1^, and apigenin at 1.6360 mg·g^−1^.

### 2.2. Differences in the Anthocyanin Compositions of Two Varieties of P. frutescens Leaves Based on Leaf Color

#### 2.2.1. Comparison of Anthocyanin Compositions in Leaves of Two Types of *P. frutescens* Based on Leaf Color

**Figure 7:**
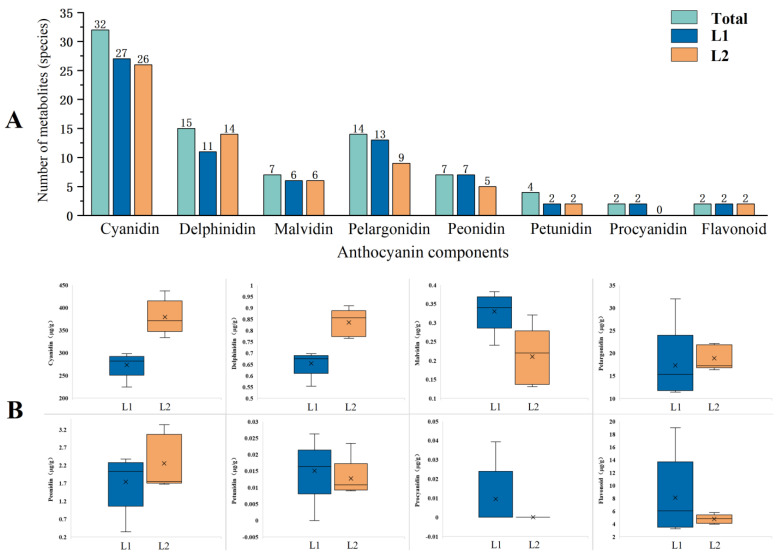
Comparison of the contents of anthocyanins in two types of *P. frutescens* leaves: (**A**) classification of anthocyanin components; (**B**) comparison of the contents of various anthocyanin components.

Leveraging fingerprinting data derived from 413 anthocyanin components, we employed gas chromatography-mass spectrometry (GC-MS) to conduct targeted analyses of anthocyanin components in *P. frutescens* leaves. A total of 83 anthocyanin compounds were identified in two varieties of *P. frutescens* leaves (Figure 7A), specifically comprising 32 types of cyanidin, 15 types of delphinidin, 7 types of malvidin, 14 types of pelargonidin, 7 types of peonidin, 4 types of petunidin, 2 types of procyanidin, and 2 types of flavonoids. Among these, *P. frutescens* leaf L1 exhibited 70 distinct anthocyanin compounds, including 27 types of cyanidin, 11 types of delphinidin, 6 types of malvidin, 13 types of pelargonidin, 7 types of peonidin, 2 types of petunidin, 2 types of procyanidin, and 2 types of flavonoids. Conversely, *P. frutescens* leaf L2 exhibited 64 distinct anthocyanin compounds, comprising 26 types of cyanidin, 14 types of delphinidin, 6 types of malvidin, 9 types of pelargonidin, 5 types of peonidin, 2 types of petunidin, and 2 types of flavonoids, with no procyanidins detected.

A quantitative analysis of the various anthocyanin compounds was performed (Figure 7B), indicating that the concentrations of cyanidin, delphinidin, pelargonidin, and peonidin in *P. frutescens* leaf L1 were 273.3487 µg·g^−1^, 0.6548 µg·g^−1^, 17.3321 µg·g^−1^, and 1.7416 µg·g^−1^, respectively. These values are lower than those observed in *P. frutescens* leaf L2, which showed concentrations reduced by 105.5796 µg·g^−1^, 0.1806 µg·g^−1^, 1.5766 µg·g^−1^, and 0.5129 µg·g^−1^. Conversely, the concentrations of malvidin, petunidin, procyanidin, and flavonoids in *P. frutescens* leaf L1 were 0.3298 µg·g^−1^, 0.0150 µg·g^−1^, 0.0996 µg·g^−1^, and 8.0647 µg·g^−1^, respectively, which are higher than those found in *P. frutescens* leaf L2 by 0.1198 µg·g^−1^, 0.0023 µg·g^−1^, 0.0096 µg·g^−1^, and 3.3145 µg·g^−1^. Targeted analysis reveals that cyanidin derivatives dominated the anthocyanin profiles (273.35–378.93 µg·g⁻^1^ in L1 vs. L2), accounting for >65% of total anthocyanins. Key differential compounds included cyanidin-3-O-glucoside (L2: 142.7 µg·g⁻^1^, 2.1× higher than L1), delphinidin-3-O-rutinoside (L1-specific), and malvidin derivatives (L2: 0.21 µg·g⁻^1^, absent in the L1). These major pigments collectively explained >80% of the leaf color variation.

#### 2.2.2. Analysis of Significantly Differentiated Metabolites of Anthocyanin Compounds in Two Varieties of *P. frutescens* Leaves Based on Leaf Color

**Figure 8:**
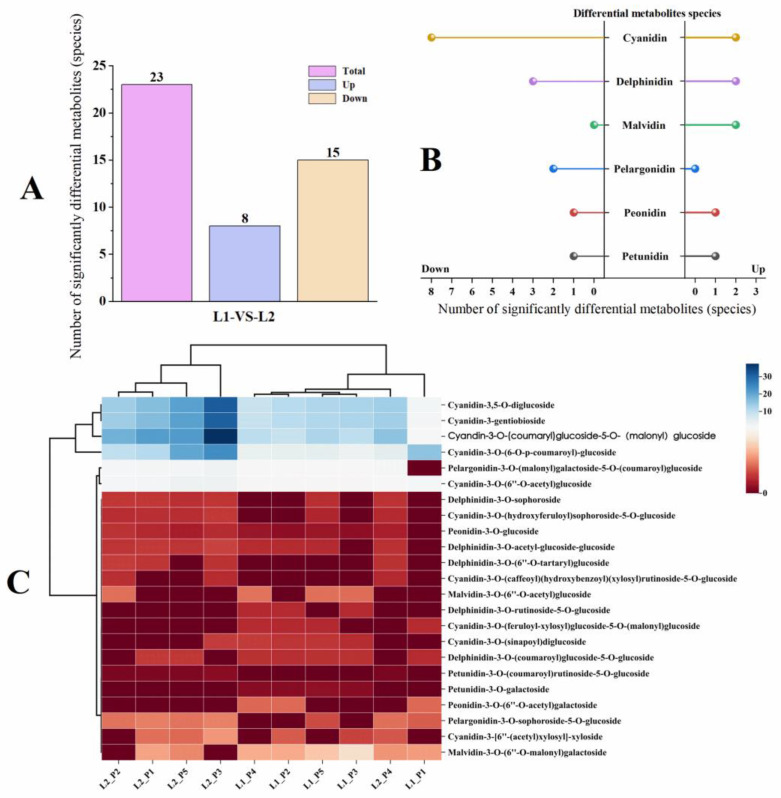
Comparison of the significantly different metabolites of the anthocyanin components in two types of *P. frutescens* leaves: (**A**) number of significantly different metabolites of the anthocyanin components; (**B**) classification of the significantly different metabolites of the anthocyanin components; (**C**) cluster analysis of two types of *P. frutescens* and significantly different metabolites of the anthocyanin components.

A differential fold change (FC) analysis was conducted on the anthocyanin compound detection data from the two varieties of *P. frutescens* leaves, selecting metabolites with an FC ≥ 2 and FC ≤ 0.5. Metabolites exhibiting a difference greater than two-fold or less than half in the experimental group compared with the control group were considered significantly different. A total of 23 significantly differentiated metabolites were identified (Figure 8A), with 8 compounds upregulated and 15 compounds downregulated in *P. frutescens* leaf L1 compared with *P. frutescens* leaf L2. These metabolites can be classified into the following six categories (Figure 8B): the 8 upregulated compounds include two types of cyanidin, two types of delphinidin, two types of malvidin, one type of peonidin, and one type of petunidin; the 15 downregulated compounds comprise eight types of cyanidin, three types of delphinidin, two types of pelargonidin, one type of peonidin, and one type of petunidin. To illustrate the relationships between the two varieties of *P. frutescens* leaves and the differences in expression of the significant metabolites, a clustering analysis was performed on these 23 differentiated anthocyanin metabolites (Figure 8C). The four origins of the L2 *P. frutescens* were clearly classified into one group, whereas the anthocyanin composition of the L2_P4 *P. frutescens* leaves was closer to that of the L1 *P. frutescens* leaves, placing them in a different group alongside the five origins of the L1 *P. frutescens*.

#### 2.2.3. Correlation Between Anthocyanin Compounds and the Yield Quality of Two Varieties of *P. frutescens* Leaves Based on Leaf Color

**Figure 9:**
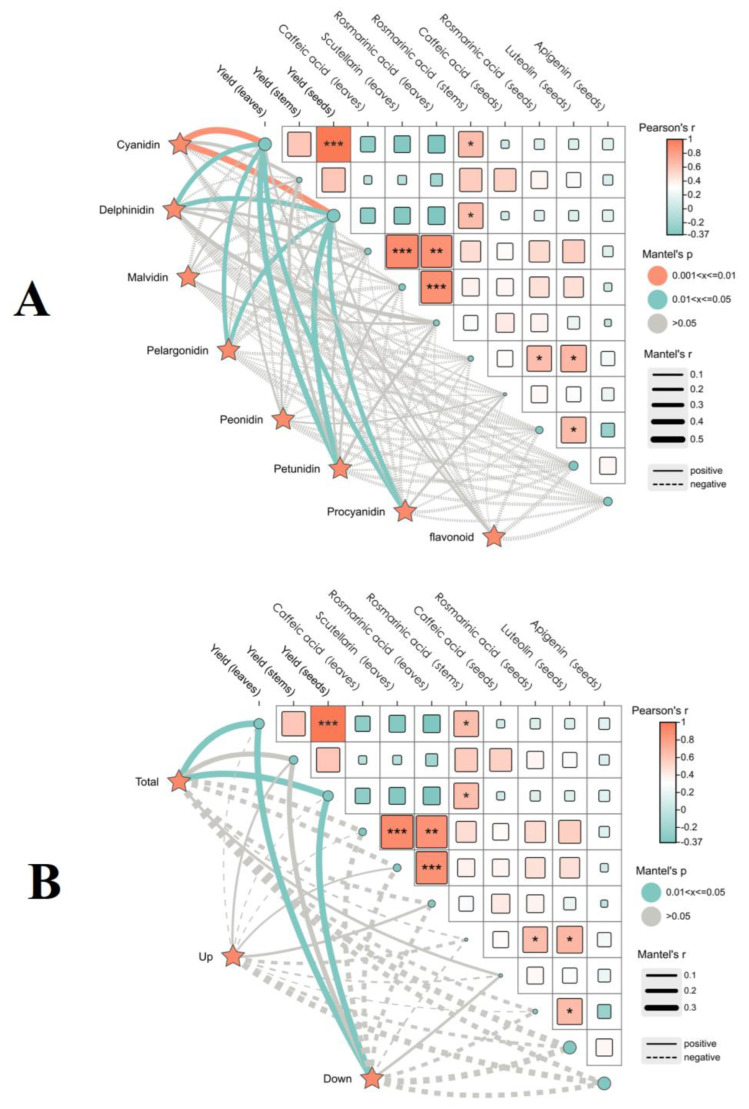
Correlation between the anthocyanin components in *P. frutescens* leaves and the yield quality of *P. frutescens*: (**A**) Mantel test between the contents of eight types of anthocyanin components in *P. frutescens* leaves and the yield quality of *P. frutescens*; (**B**) Mantel test between the contents of the significantly different metabolites of the anthocyanin components in two types of *P. frutescens* leaves and the yield quality of *P. frutescens*. * Denotes the significance level: *** *p* < 0.001, ** *p* < 0.01, * *p* < 0.05.

A Mantel test was performed to analyze the correlation between the anthocyanin components in the two varieties of *P. frutescens* leaves and the yield and quality indices of *P. frutescens* leaves, stems, and seeds (Figure 9), utilizing Pearson correlation analysis. The contents of cyanidin derivatives showed a highly significant positive correlation with the yields of *P. frutescens* leaves and seeds (*p* < 0.01) (Figure 9A). Furthermore, the contents of delphinidin, pelargonidin, petunidin, and procyanidins were significantly positively correlated with the yields of *P. frutescens* leaves and seeds (*p* < 0.05). Although various anthocyanin contents exhibited correlations with other yield-quality indices, these did not reach significance levels. Significantly differing metabolites of the anthocyanin compounds from the two varieties were positively correlated with the yields of *P. frutescens* leaves and seeds (*p* < 0.05) (Figure 9B). While the upregulated significantly differing metabolites exhibited correlations with the yield-quality indices, these did not achieve significance; conversely, the downregulated significantly differing metabolites showed significant positive correlations with the yields of *P. frutescens* leaves and seeds (*p* < 0.05).

### 2.3. Differences in Volatile Components of Two Varieties of P. frutescens Seeds Based on Leaf Color

#### 2.3.1. Comparison of Volatile Component Contents in Two Varieties of *P. frutescens* Seeds Based on Leaf Color

**Figure 10:**
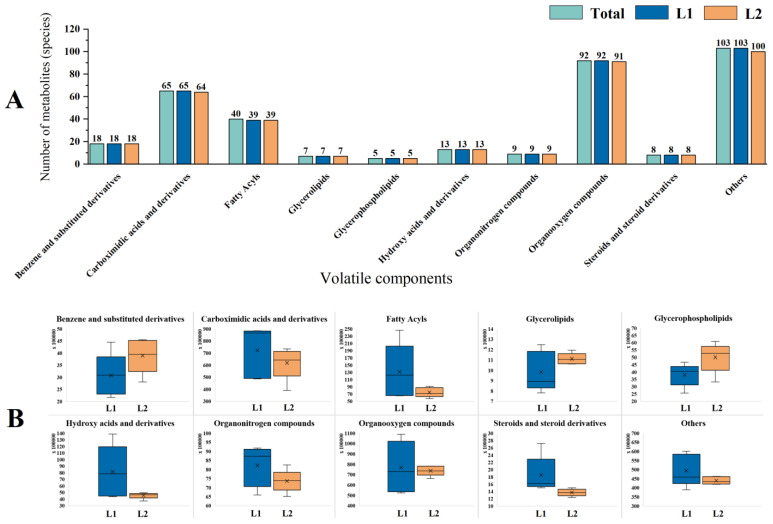
Comparison of the contents of volatile components in two types of *P. frutescens* seeds: (**A**) classification of volatile components; (**B**) comparison of the contents of various volatile components. The calculation method for the vertical coordinate was as follows: peak area of the detected substance/total peak area × average value of total peak area for all substances.

A total of 360 volatile components were identified in the two varieties of *P. frutescens* seeds (Figure 10A), comprising 18 types of benzene and substituted derivatives, 65 types of carboxylic acids and derivatives, 40 types of fatty acyls, 7 types of glycerolipids, 5 types of glycerophospholipids, 13 types of hydroxy acids and derivatives, 9 types of organonitrogen compounds, 92 types of organooxygen compounds, 8 types of steroids and steroid derivatives, and 103 additional components. *P. frutescens* seed L1 contained 359 volatile components, including 18 types of benzene and substituted derivatives, 65 types of carboxylic acids and derivatives, 39 types of fatty acyls, 7 types of glycerolipids, 5 types of glycerophospholipids, 13 types of hydroxy acids and derivatives, 9 types of organonitrogen compounds, 92 types of organooxygen compounds, 8 types of steroids and steroid derivatives, and 103 additional components. *P. frutescens* seed L2 contained 354 volatile components, comprising 18 types of benzene and substituted derivatives, 64 types of carboxylic acids and derivatives, 39 types of fatty acyls, 7 types of glycerolipids, 5 types of glycerophospholipids, 13 types of hydroxy acids and derivatives, 9 types of organonitrogen compounds, 91 types of organooxygen compounds, 8 types of steroids and steroid derivatives, and 100 additional components.

A quantitative analysis of the various volatile components was performed (Figure 10B). In *P. frutescens* seed L1, the concentrations of carboxylic acids and derivatives, fatty acyls, hydroxy acids and derivatives, organonitrogen compounds, organooxygen compounds, and steroids and steroid derivatives, as well as other volatile components, were, respectively, higher than those in *P. frutescens* seed L2 by 16.62%, 76.10%, 78.79%, 11.65%, 4.18%, 35.00%, and 12.47%. Conversely, the concentrations of benzene and substituted derivatives, glycerolipids, and glycerophospholipids were lower in *P. frutescens* seed L2 by 21.00%, 11.45%, and 23.74%, respectively. Focusing on pharmacologically relevant volatiles, perillaldehyde (L1: 12.4%, 1.8× higher than L2) and rosmarinic acid derivatives (L2: 8.7%) emerged as dominant markers. Notably, the L1 seeds showed elevated levels of α-linolenic acid (23.1% of total fatty acyls), while the L2 accumulated apigenin-linked phenolics (6.9% of organooxygen compounds). These key metabolites aligned with their distinct medicinal applications (anti-inflammatory vs. antioxidant).

#### 2.3.2. Analysis of Differential Metabolites of Volatile Components in Two Varieties of *P. frutescens* Seeds Based on Leaf Color

**Figure 11:**
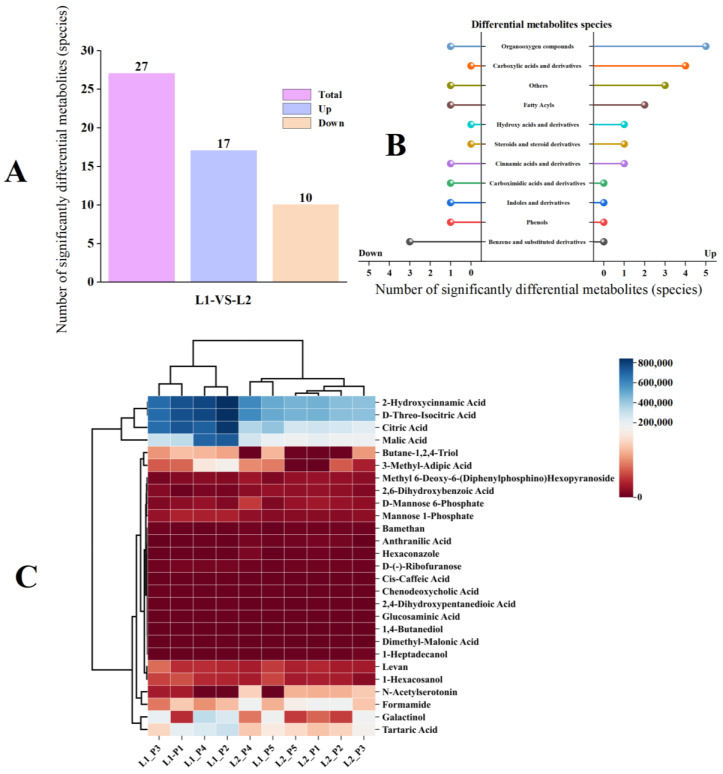
Comparison of the significantly different metabolites of the volatile components in two types of *P. frutescens* seeds: (**A**) number of significantly different metabolites of volatile components; (**B**) classification of significantly different metabolites of the volatile components; (**C**) cluster analysis of two types of *P. frutescens* and the significantly different metabolites of the volatile components.

Based on the comprehensive detection data of the volatile components in the two varieties of *P. frutescens* seeds, the parameters, such as the *p*-value, fold change, and VIP, were computed, with metabolites exhibiting a *p*-value < 0.05 and VIP > 1 considered significantly different. A total of 27 significantly different metabolites were identified (Figure 11A), with 17 compounds upregulated and 10 compounds downregulated in *P. frutescens* seed L1 compared with *P. frutescens* seed L2. These metabolites can be categorized into 11 classes (Figure 11B). Among the 17 upregulated compounds, there are five types of organic oxides, four types of carboxylic acids and derivatives, two types of fatty acyls, one type of hydroxy acid and derivatives, one type of steroid and its derivatives, one type of cinnamic acid and its derivatives, and three other substances. The 10 downregulated compounds include one type of organic oxide, one type of fatty acyl, one type of cinnamic acid and its derivatives, one type of carboxylic acid and derivatives, one type of indole and its derivatives, one type of phenolic compound, three types of benzene and substituted derivatives, and one additional substance. A clustering analysis was conducted on the 27 significantly different volatile metabolites from the two varieties of *P. frutescens* seeds originating from different regions (Figure 11C). The four origins of the L1 *P. frutescens* were distinctly classified into one group, while the four origins of the L2 *P. frutescens* formed another distinct group. However, the volatile components of the L1_P5 and L2_P4 *P. frutescens* seeds were similar and clustered together, resembling those of the L2 *P. frutescens* seeds.

#### 2.3.3. Correlation Between Volatile Components of Two Leaf Colors of *P. frutescens* and the Yield Quality of *P. frutescens*

**Figure 12:**
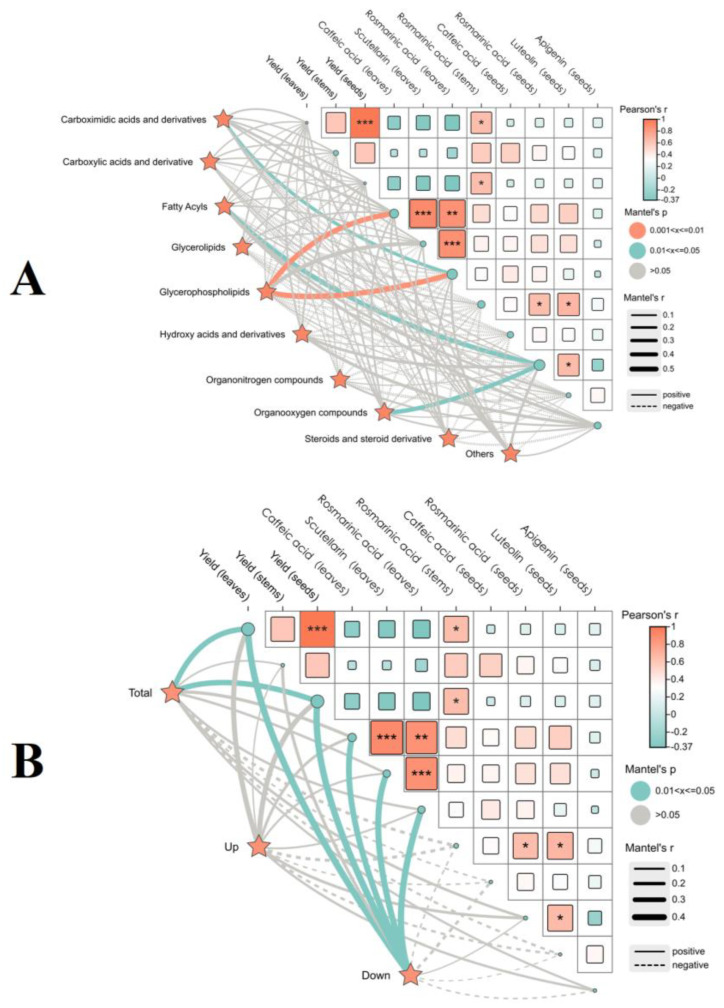
Correlations between the volatile components in two types of *P. frutescens* seeds and the yield quality of *P. frutescens*: (**A**) Mantel test between the contents of ten types of the volatile components in *P. frutescens* seeds and the yield quality of *P. frutescens*; (**B**) Mantel test between the contents of significantly different metabolites of the volatile components in two types of *P. frutescens* seeds and the yield quality of *P. frutescens*. * Denotes the significance level: *** *p* < 0.001, ** *p* < 0.01, * *p* < 0.05.

A Mantel test was conducted to assess the correlations between the volatile components of two types of *P. frutescens* seeds and the yield and quality indicators of *P. frutescens* leaves, stems, and seeds (Figure 12), employing Pearson correlation analysis. The glycerophospholipid contents among the volatile components of the *P. frutescens* seeds exhibited highly significant positive correlations with the levels of chlorogenic acid and rosmarinic acid in *P. frutescens* leaves (*p* < 0.01) (Figure 12A). The levels of benzene and substituted derivatives demonstrated a significant positive correlation with rosmarinic acid content in *P. frutescens* leaves (*p* < 0.01). Additionally, the contents of fatty acyls and organic oxidants displayed a significant positive correlation with the rosmarinic acid content in *P. frutescens* seeds (*p* < 0.01). The significantly different metabolites in the volatile components of the two types of *P. frutescens* seeds were positively correlated with the yields of *P. frutescens* leaves and seeds (*p* < 0.05) (Figure 12B). Although correlations existed between the upregulated significantly different metabolites and the yield quality indicators of *P. frutescens*, these correlations were not statistically significant. Conversely, the downregulated significantly different metabolites exhibited significant positive correlations with the yields of *P. frutescens* leaves and seeds, as well as with the levels of chlorogenic acid, baicalin, and rosmarinic acid in *P. frutescens* leaves (*p* < 0.05).

### 2.4. Correlation Between the Anthocyanin Components of P. frutescens Leaves and the Volatile Components of P. frutescens Seeds

**Figure 13:**
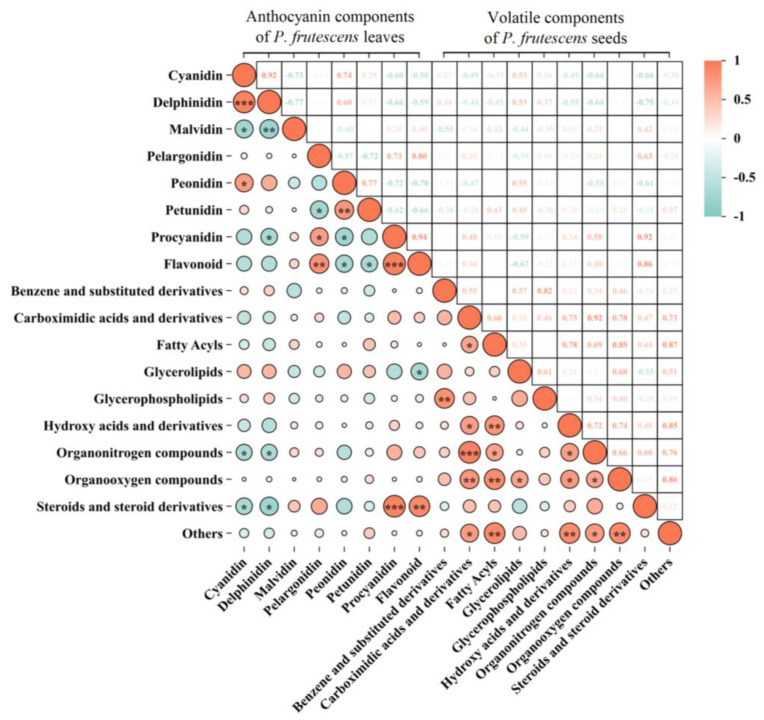
Correlations between the anthocyanin components in *P. frutescens* leaves and volatile components in *P. frutescens* seeds. * Denotes the significance level: *** *p* < 0.001, ** *p* < 0.01, * *p* < 0.05.

A Pearson correlation analysis was conducted to evaluate the contents of eight types of anthocyanin components in *P. frutescens* leaves and the contents of ten types of volatile components in *P. frutescens* seeds (Figure 13).

Correlations exist among the levels of anthocyanin components in *P. frutescens* leaves. The concentration of delphinidin shows a highly significant positive correlation with cyanidin levels (*p* < 0.001), a significant negative correlation with malvidin levels (*p* < 0.05), and a significant positive correlation with paeonidin levels (*p* < 0.05). Additionally, the delphinidin concentration is negatively correlated with both malvidin (*p* < 0.01) and proanthocyanidin levels (*p* < 0.05). Geranium levels exhibit a significant negative correlation with petunidin (*p* < 0.05), a significant positive correlation with proanthocyanidin (*p* < 0.05), and a highly significant positive correlation with flavonoid content (*p* < 0.01). Paeonidin levels demonstrate a highly significant positive correlation with petunidin (*p* < 0.01) and significant negative correlations with both proanthocyanidin and flavonoid levels (*p* < 0.05). Furthermore, the petunidin levels reflect a significant negative correlation with the flavonoid content (*p* < 0.05), whereas the proanthocyanidin levels show a highly significant positive correlation with the flavonoid content (*p* < 0.001).

Correlations exists among the levels of volatile components in *P. frutescens* seeds. The concentration of benzene and substituted derivatives exhibits a highly significant positive correlation with glycerophospholipid levels (*p* < 0.01). Additionally, the content of carboxylic acid and derivatives demonstrates significant positive correlations with fatty acyls, hydroxy acids and derivatives, and other components (*p* < 0.05), along with highly significant positive correlations with organonitrogen compounds (*p* < 0.001) and organooxygen compounds (*p* < 0.01). Fatty acyl levels are also highly significantly positively correlated with hydroxy acids and derivatives, organooxygen compounds, and other components (*p* < 0.01), as well as show a significant positive correlation with organonitrogen compounds (*p* < 0.05). Glyceride concentrations reveal a significant positive correlation with organooxygen compounds (*p* < 0.05). Hydroxy acid levels and their derivatives exhibit significant positive correlations with organonitrogen and organooxygen compounds (*p* < 0.05), as well as a highly significant positive correlation with other components (*p* < 0.01). Furthermore, organonitrogen compounds show significant positive correlations with organooxygen compounds and other components (*p* < 0.05), while organooxygen compounds also demonstrate highly significant positive correlations with other components (*p* < 0.01).

A correlation exists between the levels of anthocyanin components in *P. frutescens* leaves and the levels of volatile components in *P. frutescens* seeds. The concentrations of delphinidin and cyanidin in *P. frutescens* leaves show significant negative correlations with the levels of organonitrogen compounds, as well as steroids and steroid derivatives, in *P. frutescens* seeds (*p* < 0.05). In contrast, the content of proanthocyanidins exhibits a highly significant positive correlation with the levels of steroids and steroid derivatives (*p* < 0.001). Furthermore, flavonoid content demonstrates a significant negative correlation with glycerolipids (*p* < 0.05) and a highly significant positive correlation with steroids and steroid derivatives (*p* < 0.01).

## 3. Discussion

### 3.1. Significant Differences in Yield and Quality Exist Between the Two Leaf Color Varieties of P. frutescens

The aboveground portions of *P. frutescens*, including the leaves, stems, and seeds, possess significant medicinal value. However, *P. frutescens* leaves exhibit various colors; generally, only those that are green on the adaxial surface and purple on the abaxial surface, as well as those that are entirely purple, are permitted for medicinal use. This study revealed significant differences in the yield and quality between the two leaf color varieties. The yields of the leaves, stems, and seeds from the green-on-adaxial and purple-on-abaxial variety are consistently higher than those from the entirely purple variety. This finding is consistent with research conducted by Su X et al. [16], which indicates that deep green leaves enhance photosynthesis and effectively improve crop yields. This advantage may be attributed to the superior physiological capabilities of the green-on-adaxial and purple-on-abaxial variety, facilitating higher photosynthetic efficiency, better overall plant growth, and increased yield [17]. The quality of leaves from the purple on both sides *P. frutescens* is superior to that of the green on the front and purple on the back variety. This observation aligns with the findings of Saini R.K. et al. [18], which indicate that important nutritional metabolites are higher in purple *P. frutescens* leaves compared with their green counterparts. Although differences exist in the quality of *P. frutescens* seeds and stems between the two leaf color varieties, these differences are minor, suggesting that leaf color is primarily associated with leaf quality. This finding corroborates the stipulation in the 2020 edition of the Pharmacopoeia of the People’s Republic of China that specifies the color of *P. frutescens* leaves but does not address the colors of *P. frutescens* seeds and stems.

### 3.2. Anthocyanin Components of P. frutescens Leaves Are Closely Related to the Yield and Quality of P. frutescens

The color of plant leaves is predominantly determined by the presence of chlorophyll and anthocyanins. Chlorophyll imparts a green hue to the leaves, whereas anthocyanins contribute to purple, red, or blue colors [19,20]. The analysis of the anthocyanin components in two types of purple *P. frutescens* leaves reveals that the total anthocyanin content in the L2 leaves was 405.8998 µg·g^−1^, representing a 34.63% increase compared with L1, with a notably significant difference in cyanidin content. This finding aligns with the research conducted by Saini R.K. et al. [18], which demonstrated that the anthocyanin content in violet-purple leaves is higher than that in green leaves of *P. frutescens*. Furthermore, a metabolite analysis revealed significant differences in anthocyanins between the two types of *P. frutescens* leaves, indicating that among the 15 anthocyanin components downregulated in L1 compared with L2, 8 were identified as cyanidin. This observation may be attributed to the higher stability and antioxidant capacity of cyanidin, rendering it less susceptible to degradation during plant growth and experimental procedures [21,22]. Cyanidin is characterized by a reddish-purple or deep purple color, whereas the other seven types exhibit colors such as red, bluish-purple, light brown, and brown. Cyanidin not only displays a more intense reddish-purple hue, but it also has the highest content among these pigments, demonstrating the greatest variability. Therefore, it can be inferred that the differences in cyanidin content are critical factors influencing the color of purple *P. frutescens* leaves [23,24,25].

The Mantel test identified significant positive correlations between the anthocyanin components in purple *P. frutescens* leaves—specifically delphinidin, cyanidin, pelargonidin, paeonidin, and petunidin—and the yields of both purple *P. frutescens* leaves and *P. frutescens* seeds. Notably, the correlation of cyanidin content was highly significant, consistent with the findings of Song S et al. [26]. The Mantel test assessing the significant differences in the anthocyanin components between the two types of purple *P. frutescens* and their yield quality indicates that the downregulated anthocyanin components in the L1 leaves are significantly positively correlated with the yields of both purple *P. frutescens* leaves and seeds. Among the 15 downregulated components, 8 are classified as cyanidin, suggesting highly significant positive correlations between cyanidin and the yields of both leaves and seeds. Furthermore, based on comparisons of the anthocyanin determinations, cyanidin content is inferred to be the most critical factor affecting the color of purple *P. frutescens* leaves. It can be concluded that considerable differences in leaf and seed yields exist between the two types of purple *P. frutescens*, and the findings of this experiment regarding significant differences in the yields of the two varieties support each other. Additionally, the experimental results indicate negative correlation trends between the anthocyanin content and the yields of leaves and seeds, suggesting that the promoting effects of individual anthocyanin components may be offset within the broader context of total metabolism [27]. Moreover, this phenomenon is also related to the antagonistic effects of plant components [28], threshold effects [29,30], and resource trade-off theory [31,32].

### 3.3. Volatile Components of P. frutescens Seeds Are Closely Related to the Yield and Quality of Purple P. frutescens

Volatile oil from *P. frutescens* seeds often represents their quality [8]. An analysis of the volatile components in the two types of *P. frutescens* seeds with different leaf colors reveals that the total volatile component content in the L1 seeds was 12.99% higher than that in the L2 seeds, along with the identification of five additional types. A total of 27 metabolites with significant differences were detected, indicating a notable disparity in the volatile components between the two types of *P. frutescens* seeds, consistent with previous research findings [11,18,33]. Organooxygen compounds represent the largest and most abundant category of volatile components in *P. frutescens* seeds. Moreover, the levels of organooxygen compounds in the L1 leaves were higher than those in L2. Of the 17 significantly different metabolites upregulated in the L1 compared with L2 leaves, five belong to the category of organooxygen compounds, which constitutes the highest proportion of these differential metabolites. This observation may be linked to differences in the antioxidant capacity associated with variations in anthocyanin content, as varying antioxidant abilities result in significant disparities in the accumulated organooxygen compounds [34,35].

The Mantel test indicated that the volatile components of *P. frutescens* seeds, including benzene and substituted derivatives, fatty acyls, glycerophospholipids, and organooxygen compounds, exhibited significant positive correlations with the quality of both purple *P. frutescens* leaves and seeds. This finding suggests potential associations between the volatile components of *P. frutescens* seeds and the quality of various medicinal parts of the plant, aligning with previous research findings [9,36]. Based on the differences in the contents of various related components between the two types of *P. frutescens* seeds, it can be inferred that the leaf and seed quality of the L2 purple *P. frutescens* is superior to that of L1, which supports the findings of this study. The results of the Mantel test concerning the significantly different metabolites in the volatile components of the two types of *P. frutescens* seeds indicate that the downregulated components in the L1 purple *P. frutescens* seeds are significantly correlated with the yield and quality of both purple *P. frutescens* leaves and seeds. These downregulated components include benzene and substitute derivatives, phenols, indoles and their derivatives, carboxylic acids and their derivatives, cinnamic acids and their derivatives, fatty acyls, organooxygen compounds, and others. Compounds, such as aromatics, alcohols, acids, and esters, in the volatile components of *P. frutescens* are significantly associated with plant growth and development. The differences in the leaf color directly affect the photosynthesis and antioxidant capacity of purple *P. frutescens* leaves, leading to variations in the accumulation of volatile components and resulting in significant correlations between the volatile components of *P. frutescens* seeds and the yield and quality of purple *P. frutescens* [9,36,37,38].

### 3.4. Accumulation of Anthocyanins in P. frutescens Leaves Is Closely Related to the Accumulation of Volatile Components in P. frutescens Seeds

The correlation analysis of the anthocyanin components in *P. frutescens* flowers with the volatile components of *P. frutescens* seeds demonstrates that the levels of proanthocyanidins and flavonoids in *P. frutescens* leaves are significantly positively correlated with the concentrations of steroids and steroid derivatives in *P. frutescens* seeds. This correlation may be attributed to the superior antioxidant capacity of proanthocyanidins and flavonoids compared with other components, particularly due to proanthocyanidins’ strong ability to scavenge free radicals and reduce oxidative stress. As a result, this antioxidant activity alleviates the inhibitory effects of oxidative stress on the metabolic processes of plants, thereby promoting the synthesis and accumulation of steroids and steroid derivatives [39,40]. The analysis results indicate that various anthocyanin components in *P. frutescens* leaves are significantly correlated with volatile components in *P. frutescens* seeds. This correlation is linked to the antioxidant properties of anthocyanins and their roles in regulating plant hormones, influencing enzyme activity, modulating compound metabolism, and participating in plant signal transduction pathways [41,42,43,44].

## 4. Materials and Methods

### 4.1. Overview of the Test Area

**Figure A2:**
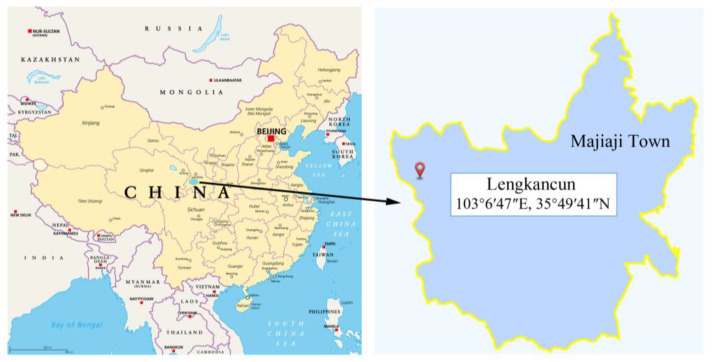
Schematic diagram of the test site.

The experiment was conducted from April to November 2024 at the demonstration base for the integration of production, education, and research related to Chinese medicinal materials at the College of Agriculture, Gansu Agricultural University, located in Langkan Village, Majiajic Town, Weiyuan County, Gansu Province (103°6′47″ E, 35°49′41″ N) (Figure A2). The average annual temperature at the experimental site is 6.1 °C, with a frost-free period of 130 days and annual precipitation of approximately 600 mm, at an elevation of 2350 m. The organic matter content of the soil in the 0–30 cm layer is 24.66 g·kg^−1^, total nitrogen is 1.3 g·kg^−1^, available phosphorus is 16.14 mg·kg^−1^, and quick-acting potassium is 112.8 mg·kg^−1^. The soil type is predominantly black loam, with the preceding crop being the Apiaceae species, Angelica.

### 4.2. Plant Materials, Reagents, and Instruments

*P. frutescens* seeds were purchased from local farmers at the end of 2023, all of which were new seeds from that year. They were identified by a researcher, Jin Xiaojun, from Gansu Agricultural University as seeds of the Lamiaceae family, *Perilla frutescens* (L.) Britt.

The standard reference substances utilized in the high-performance liquid chromatography included luteolin (batch number: B20888, purity ≥ 98%), scutellarin (batch number: B21478, purity ≥ 98%), rosmarinic acid (batch number: B20862, purity ≥ 98%), caffeic acid (batch number: B20660, purity ≥ 98%), and apigenin (batch number: B20981, purity ≥ 98%), all of which were procured from Shanghai Yuanye Biotechnology Co., Ltd. (Shanghai, China). The analysis of anthocyanin components in *P. frutescens* leaves was carried out by Wuhan Matver Biotechnology Co., Ltd. (Wuhan, China), while the evaluation of volatile components in *P. frutescens* seeds was conducted by Shanghai Luming Biotechnology Co., Ltd. (Shanghai, China). The instruments employed included the Agilent 1260 II high-performance liquid chromatograph (Agilent Technologies, Santa Clara, CA, USA), Qtrap 6500+ liquid chromatography-tandem mass spectrometer (Shanghai Aibocai Analysis Instrument Trading Co., Ltd. (Shanghai, China)), Agilent 7890B-5977A gas chromatography-mass spectrometer (Agilent Technologies, Santa Clara, CA, USA), TGL18M centrifuge (Hunan Kaida Scientific Instrument Co., Ltd. (Changsha, China)), and T6 New Century UV-Vis spectrophotometer (Beijing PUXI General Instrument Co., Ltd. (Beijing, China)), among others.

### 4.3. Experimental Design

This experiment employed a two-factor randomized block design, with nitrogen fertilizer application as the primary factor and *P. frutescens* leaf color as the secondary factor. *P. frutescens* was categorized based on leaf color into the following two varieties: green-backed purple *P. frutescens* (L1) and double-sided purple *P. frutescens* (L2). Both varieties were sourced from the same five production areas, designated as P1 to P5 (Table A1). A total of 10 treatments were established, with three replicates, resulting in 30 experimental plots. Sowing was uniformly conducted on 10 May 2024, with a plant spacing of 15 cm and a row spacing of 25 cm. A black film was applied one week in advance to retain moisture and warmth, with hole planting and a soil cover depth of 3 to 5 mm. Each plot measured 1 m × 9 m (9 m^2^) and was bordered by a protective row 1.5 m wide. Except for fertilization, all other field management practices were consistent across the plots.

### 4.4. Determination of Yield and Quality

#### 4.4.1. Yield Determination

In each plot, 15 *P. frutescens* plants were selected for study. *P. frutescens* leaves and stems were harvested at the appropriate time for weight measurement. After the seeds reached full maturity, they were collected to determine weight and thousand-seed weight. The yield per hectare was calculated by integrating the measurement results with planting density.

*P. frutescens* Leaf Yield: Fresh leaves, measuring 8 to 12 cm in width, were harvested at regular intervals prior to the flowering of *P. frutescens* plants. The fresh weight was recorded using a precision scale with a sensitivity of 0.01 g, and the dry weight was determined after complete shade drying.

*P. frutescens* Stem Yield: The aerial parts of the *P. frutescens* plants were harvested prior to flowering. All leaves were removed, and the remaining stems were completely shade-dried. The dry weight was determined using a precision scale with a sensitivity of 0.01 g.

*P. frutescens* Seed Yield: Seeds were harvested upon reaching full maturity and then shade-dried completely. The weight was determined using a precision scale with a sensitivity of 0.01 g.

Thousand-Seed Weight of *P. frutescens*: The thousand-seed weight was determined using an electronic balance with a sensitivity of 0.0001 g.

#### 4.4.2. Quality Determination

Sample Preparation: *P. frutescens* leaves, stems, and seeds were thoroughly dried, then ground and sieved. A precise amount of either 1 g of *P. frutescens* leaves, 1 g of *P. frutescens* stems, or 0.5 g of *P. frutescens* seed powder was placed in a 25 mL volumetric flask and brought to volume with 70% methanol. The mixture was weighed and subjected to ultrasonic extraction for 40 min (250 W, 40 kHz, 40 °C). After cooling to room temperature, the mixture was reweighed, and any weight loss was compensated with additional 70% methanol. The solution was then centrifuged at 9000 rpm for 10 min, and the supernatant was filtered through a 0.45 μm organic microporous membrane to obtain the final solution.

Investigation of Linear Relationships: A precise quantity of the standard reference material was weighed to prepare a series of mixed standard solutions. These solutions were filtered through a 0.45 μm organic microporous membrane and analyzed under the specified chromatographic conditions. The sample’s mass concentration (X in µg·mL^−1^) was plotted on the x-axis, while the peak area (Y) was plotted on the y-axis to construct the standard curve for each component. The regression equations and correlation coefficients were calculated, with the results presented in Table 1.

**Figure 14:**
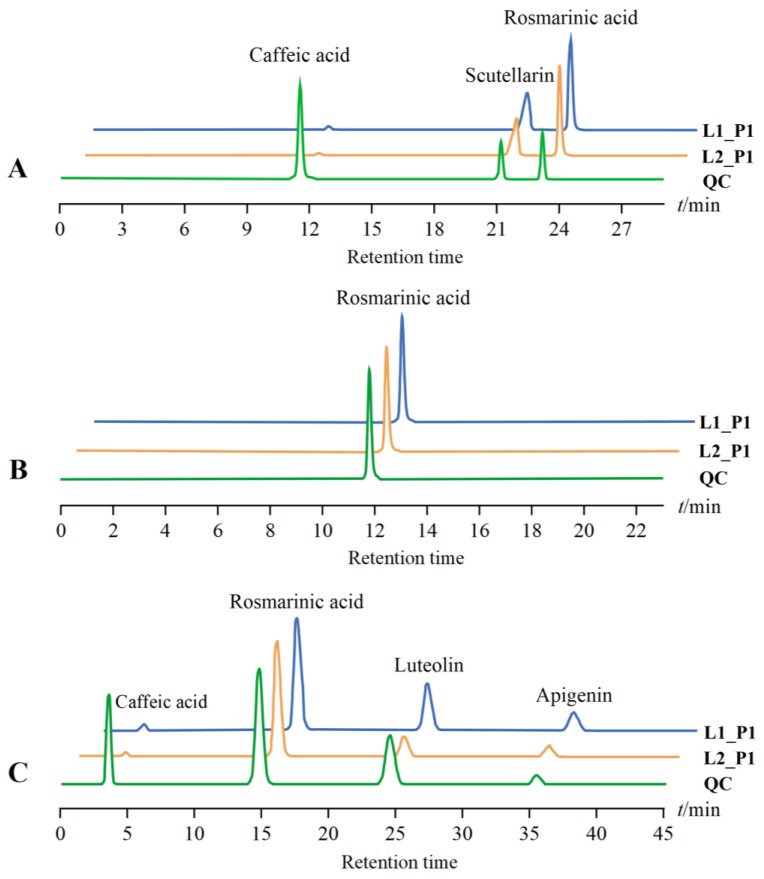
Chromatograms of the tested *P. frutescens* samples and mixed control samples: (**A**) *P. frutescens* leaves; (**B**) *P. frutescens* stems; (**C**) *P. frutescens* seeds. L: different leaf color treatments; P: different cultivation locations; QC: results of the sample injection of the mixed standard.

Determination of Caffeic Acid, Scutellarin, and Rosmarinic Acid Content in *P. frutescens* Leaves: High-performance liquid chromatography (HPLC) was utilized (Figure 14A). The chromatographic conditions were as follows: column: Waters Symmetry^®^ C18 (250 mm × 4.6 mm, 5 µm); mobile phase: methanol (A)—0.1% phosphoric acid (B), utilizing gradient elution (0 to 10 min, 25% → 30% A; 10 to 13 min, 30% → 38% A; 13 to 15 min, 38% → 45% A; 15 to 22 min, 45% → 50% A; 22 to 24 min, 50% → 25% A; 24 to 29 min, 25% A); flow rate: 1 mL·min^−1^; injection volume: 10 µL; column temperature: 30 °C; detection wavelength: 320 nm.

Determination of Rosmarinic Acid Content in *P. frutescens* Stems: High-performance liquid chromatography (HPLC) was utilized (Figure 14B). The chromatographic conditions were as follows: column: Waters Symmetry^®^ C18 (250 mm × 4.6 mm, 5 µm); mobile phase: methanol and acetonitrile = 1:1 ratio (A)—0.5% acetic acid (B), utilizing gradient elution (0 to 3 min, 10% → 40% A; 3 to 16 min, 40% → 50% A; 16 to 18 min, 50% → 10% A; 18 to 23 min, 10% A); flow rate: 0.8 mL·min^−1^; injection volume: 10 µL; column temperature: 35 °C; detection wavelength: 254 nm.

Determination of Caffeic Acid, Rosmarinic Acid, Luteolin, and Apigenin Content in *P. frutescens* Seeds: High-performance liquid chromatography (HPLC) was utilized (Figure 14C). The chromatographic conditions were as follows: column: Waters Symmetry^®^ C18 (250 mm × 4.6 mm, 5 µm); mobile phase: methanol: acetonitrile = 1:1 (A)—0.5% acetic acid (B), utilizing gradient elution (0 to 5 min, 10% → 40% A; 5 to 35 min, 40% → 60% A; 35 to 40 min, 60% → 10% A; 40 to 45 min, 10% A); flow rate: 0.8 mL·min^−1^; injection volume: 10 µL; column temperature: 35 °C; detection wavelength: 254 nm.

### 4.5. Analysis of Metabolic Products

#### 4.5.1. Analysis of Anthocyanin Components in *P. frutescens* Leaves

On 25 August 2024, ten random samples of *P. frutescens* plants were collected from each neighborhood, focusing on uniform-sized leaves from the upper portions of the plants. The samples were vacuum-freeze-dried and ground into powder using a ball mill (30 Hz, 1.5 min). Fifty milligrams of the powdered sample was then dissolved in 500 µL of an extraction solution composed of 50% methanol and 0.1% hydrochloric acid. After vortex mixing, ultrasonic extraction was carried out for 5 min, followed by centrifugation (12,000 rpm, 4 °C, 3 min). The supernatant was carefully collected, and this procedure was repeated. The two supernatant fractions were combined and filtered through a 0.22 µm micropore filter for the LC-MS metabolomics analysis. The quantitative and qualitative analyses of the 413 components, including anigozanthos, delphinidin, malvidin, paeonidin, centaureidin, geranium, proanthocyanidin, and flavonoids, were performed using ultra-high-performance liquid chromatography (UPLC) coupled with tandem mass spectrometry (MS). The chromatographic conditions were established as follows: column: Acquity BEH C18 (100 mm × 2.1 mm, 1.7 µm); mobile phase: methanol (A) and 0.5% formic acid (B); gradient elution (0 to 6 min, 5% → 50% A; 6 to 12 min, 50% → 95% A; 12 to 14 min, 95% A; 14 to 16 min, 5% A); flow rate: 0.35 mL·min^−1^; injection volume: 2 µL; column temperature: 40 °C. The mass spectrometry conditions included an electrospray ionization (ESI) source temperature of 550 °C, a mass spectrometry voltage of 5500 V in the positive ion mode, and an auxiliary gas flow (CUR) of 35 psi. In the Q-Trap 6500+, each ion pair was scanned and detected based on the optimized declustering potential (DP) and collision energy (CE).

#### 4.5.2. Analysis of Volatile Components in *P. frutescens* Seeds

After fully maturing, *P. frutescens* seeds were harvested, air-dried, and cleaned. A total of 60 mg of *P. frutescens* seeds was weighed and placed into a 1.5 mL centrifuge tube, into which two small steel balls and 600 µL of a methanol–water solution (V:V = 7:3, containing a mixed internal standard at 4 µg·mL^−1^) were added. The tube was pre-cooled in a −40 °C freezer for 2 min, then ground in a mill at 60 Hz for 2 min. Ultrasonic extraction was performed in an ice-water bath for 30 min, after which the mixture was left to stand at −40 °C for 2 h before centrifugation (13,000 rpm, 4 °C, 10 min). A total of 150 µL of the supernatant was transferred to a glass derivatization vial, and the samples were dried using a centrifugal concentrator. Subsequently, 80 µL of a methoxyamine hydrochloride–pyridine solution (15 mg·mL^−1^) was added to the derivatization vial, and the reaction was incubated in a shaker at 37 °C for 60 min to facilitate oximation. After removing the sample, 50 µL of BSTFA (N, O-bis (trimethylsilyl) trifluoroacetamide) derivatization reagent and 20 µL of n-hexane were added, along with 10 internal standards (C8/C9/C10/C12/C14/C16/C18/C20/C22/C24, all prepared in chloroform) at 10 µL each. The reaction was conducted at 70 °C for 60 min. The sample was then allowed to stand at room temperature for 30 min before undergoing GC-MS metabolomics analysis. The gas chromatography conditions were as follows: column: Agilent DB-5MS capillary column (30 m × 0.25 mm, 0.25 µm); carrier gas: high-purity helium (purity not less than 99.999%); temperature program: the initial column temperature was set at 60 °C and held for 0.5 min; ramped to 125 °C at a rate of 8 °C·min^−1^; ramped to 210 °C at 8 °C·min^−1^; ramped to 270 °C at 15 °C·min^−1^; ramped to 305 °C at 20 °C·min^−1^ and held for 5 min; flow rate: 1.0 mL·min^−1^; injection port temperature: 260 °C; injection volume: 1 µL, non-split injection; solvent delay: 5 min. The mass spectrometry conditions included an electron impact ionization (EI) source with an ion source temperature of 230 °C, a quadrupole temperature of 150 °C, and an electron energy of 70 eV. Scanning was performed in the full-scan mode (SCAN), with a mass scanning range of m/z 50–500.

### 4.6. Data Analysis

Data regarding the yield of *P. frutescens* and the content of effective components are expressed as the mean ± standard deviation. Data analysis was performed using IBM SPSS Statistics 26.0 (IBM Corp, Armonk, NY, USA), and relevant images were created using OriginPro 2021 (OriginLab, Northampton, MA, USA) and Adobe Illustrator 2023 (Adobe Inc., San Jose, CA, USA). Metabolite images were plotted using Excel 2019 (Microsoft Corporation, Redmond, WA, USA) and OriginPro 2021 (OriginLab, Northampton, MA, USA). Pearson correlation analysis and Mantel tests were conducted using Chiplot (www.chiplot.online/ accessed on 8 December 2025).

## 5. Conclusions

This study provides a comprehensive comparison of the yields, quality, and metabolic profiles between green-backed purple (L1) and double-sided purple (L2) *P. frutescens* cultivated across diverse geographical origins. Key findings reveal significant trade-offs between biomass production and bioactive compound accumulation in the two varieties. L1 demonstrated superior agronomic performance, with leaf, stem, and seed yields exceeding L2 by 43.1%, 36.6%, and 32.3%, respectively. In contrast, L2 exhibited enhanced medicinal quality, characterized by 34.6% higher total anthocyanins in leaves and elevated levels of caffeic acid, scutellarin, and rosmarinic acid in leaves/seeds. Metabolic profiling further identified cyanidin derivatives as pivotal contributors to leaf coloration and yield correlations, while organooxygen compounds in seeds emerged as key quality markers.

Notably, the Mantel test revealed a significant negative correlation between anthocyanin accumulation in the leaves’ and seeds’ volatile components, suggesting resource allocation trade-offs between vegetative and reproductive metabolic pathways. These findings underscore the importance of leaf color as a phenotypic marker for breeding programs aimed at optimizing either biomass production (L1) or phytochemical richness (L2). The geographical origin analysis highlighted P2 (L1) and P4 (L2) as optimal cultivation sites for maximizing yield and quality, respectively.

This work advances the practical selection of *P. frutescens* varieties for targeted applications—L1 for industrial-scale biomass production and L2 for pharmaceutical/nutraceutical uses. Future studies should explore genetic regulators of the observed metabolic trade-offs and validate these patterns under varying environmental conditions to enhance cultivar adaptability.

## Figures and Tables

**Table 1:** Results of the linear relationship investigation.

Ingredient to Be Tested	Regression Equations	Correlation Coefficient	Linear Range (μg·mL^−1^)
Caffeic acid (Leaves)	Y = 62.65X − 35.579	0.9995	2~12
Scutellarin (Leaves)	Y = 23.286X − 4.4053	0.9995	2~12
Rosmarinic acid (Leaves)	Y = 23.145X + 8.0847	0.9992	2~12
Rosmarinic acid (Stems)	Y = 14.639X − 9.3247	0.9997	2~12
Caffeic acid (Seeds)	Y = 21.012X + 2.9191	0.9996	2.5~37.5
Rosmarinic acid (Seeds)	Y = 11.386X − 19.138	0.9993	10~150
Luteolin (Seeds)	Y = 57.498X − 1.9701	0.9992	1~15
Apigenin (Seeds)	Y = 20.444X + 0.7979	0.9992	0.5~7.5

## Data Availability

Data are contained within the article.

## Appendix A

**Table A1:** Origin and types of *P. frutescens*.

Leaf Color Classification	Origin				
	P1	P2	P3	P4	P5
Green-backed purple *P. frutescens* (L1)	Heilongjiang	Fujian	Hunan	Hebei	Gansu
Double-sided purple *P. frutescens* (L2)	Heilongjiang	Fujian	Hunan	Hebei	Gansu
